# Paediatric Heart Transplantation in Resource-Limited Settings: A Silent Crisis

**DOI:** 10.31083/RCM47034

**Published:** 2026-02-04

**Authors:** Janett Francis, Andra Dobromirescu, Jeevan Francis

**Affiliations:** ^1^Department of Cardiothoracic Surgery, Aberdeen Royal Infirmary, AB25 2ZN Aberdeen, UK; ^2^Department of Cardiac Surgery, Guy's and St Thomas' NHS Foundation Trust, SE1 7EH London, UK

## 1. Background 

Congenital heart disease (CHD) remains the leading cause of death from birth defects worldwide, with over 250,000 infant deaths in 2021 alone [1]. CHD is the leading etiology requiring transplantation in children, accounting for around 50% of all pediatric heart transplants [2]. For children with end-stage heart failure, heart transplantation represents the only definitive therapy. Globally, more than 6000 heart transplants are performed each year [3], with approximately 600 in pediatric patients [4]. Yet, the overwhelming majority occur in high-income countries (HICs), with more than 75% of these taking place in the United States in the past 10 years [2]. According to the International Organ Transplant Registry, more than 80% of pediatric heart transplants take place in North America, about 15% take place in Europe, and only 3.2% take place across all other World Health Organization (WHO) regions. This global disparity, derived from the data collected from 1992 to 2024, emphasises that pediatric heart transplant activity remains uneven worldwide and is concentrated in HIC [5]. In contrast, children in countries with limited resources face a grim reality: advanced therapies, including transplantation, are rarely available, and mortality rates remain unacceptably high. This silent crisis highlights the urgent need to expand pediatric heart transplantation services globally to provide life-saving care for children.

## 2. The Unmet Need

Children in low- and middle-income countries (LMICs) experience disproportionately high mortality and morbidity from conditions that are treatable in wealthier nations [6]. For example, in HICs, the prognosis for CHD surgery is significantly better due to advancements in surgical care, whereas in LMICs, resource limitations continue to contribute to poorer survival [7]. In Africa, pediatric heart transplants are still largely unavailable, with only infrequent cases reported. As of 2022, most African countries have no sustainable pediatric transplant programs, although Tunisia was a rare example, having reported a cardiac transplant that year [8]. This situation is particularly dire for pediatric patients, who have the highest waitlist mortality among all transplant candidates and solid organ recipients. Contributing factors include limited donor availability, small body sizes that restrict suitable grafts, and the presence of complex congenital comorbidities [2].

## 3. Barriers to Pediatric Heart Transplantation 

The obstacles to establishing sustainable pediatric transplantation programs in LMICs are multifactorial:

- Infrastructure and workforce shortages: Across many LMICs, there are fewer than 0.05 pediatric cardiac surgeons per million children, with over 70 countries globally having no reported pediatric cardiac surgeons [9]. Only a handful of centers have the ability to provide the comprehensive transplantation services needed, including extracorporeal membrane oxygenation (ECMO), ventricular assist devices (VADs), and long-term monitoring of immunosuppression.
- Donor scarcity: Pediatric organ donation is uncommon. Cultural barriers [10], lack of pediatric donation initiatives, and logistical challenges in organ transport, often relying on commercial flights over vast distances, make timely transplantation nearly impossible [11]. Because pediatric organ donations are infrequent, many patients die while waiting for a suitable organ, and when donations do occur, there are often no immune-compatible recipients available to receive them [12].
- Financial barriers: Transplantation is among the most resource-intensive surgical interventions. In settings where adult cardiac surgery is underfunded, governments and health systems struggle to support the enormous costs of transplantation, mechanical support, and long-term immunosuppressive therapy [13]. Additionally, the financial burden on the family of a patient is significant. For example, in Lebanon, patients have to pay $ 130,000 out-of-pocket for VAD treatment [14]. Many families in LMIC simply cannot afford this costly treatment.
- Postoperative challenges: Follow-up care for transplant recipients is difficult and resource-intensive, particularly in healthcare systems that are already overstretched. Key issues include infection control, rejection, and adherence to immunosuppression [15]. According to a multi-institutional study, more than 40% of pediatric heart transplant patients develop at least one infection, with many of these patients experiencing multiple infections [16]. In another study evaluating rejection rates, it was found that although rejection rates are decreasing, there is still a significant number of children affected. Between 2008 and 2012, 22% of children experienced rejection within the first year following transplantation [17]. This ongoing difficulty of maintaining optimal postoperative care highlights a barrier to pediatric heart transplantation.
- Sparse data on outcomes of pediatric heart transplants: To improve the development of pediatric heart transplants, it is necessary to have registries, outcome reporting, and collaborative research. Such initiatives will allow for more accurate identification of best practices in resource-limited settings.

## 4. Bridging the Gap: Mechanical Circulatory Support 

Mechanical circulatory support (MCS), particularly VADs and ECMO, has revolutionized pediatric transplantation in HICs by bridging critically ill children to transplantation. In a recent report, over 40% of heart transplant patients in HICs were bridged to transplant with the use of VADs. A pediatric heart transplant study (PHTS) reported that the survival percentage for those on VADs was substantially greater than that of those bridged with ECMO [18]. ECMO is typically indicated for short-term stabilization, while VADs offer more long-term support for patients awaiting transplantation.

In another study, including 2777 pediatric patients who required heart transplants, 22% required MCS, with almost 70% of those patients requiring a VAD, and the other 30% requiring ECMO. Post-transplant survival rates for those with VADs were comparable to those who needed direct transplantation, whereas ECMO patients had less favourable outcomes. This was evident as the 1-year after transplantation survival rates for direct transplantation, bridging through VAD, and ECMO were 91%, 90% and 61% respectively [19]. Nearly one-third of pediatric recipients in the International Society for Heart and Lung Transplantation (ISHLT) registry are on MCS at the time of transplant [20]. VADs have decreased waitlist mortality and yield post-transplant survival comparable to non-VAD recipients [21].

However, in LMICs, VADs remain almost entirely inaccessible due to cost and infrastructure barriers. For example, the cost of implanting a VAD in Lebanon is $ 150,000, with over 85% of the costs coming out-of-pocket from the families of the affected child [14]. In LMIC, the supply chain and manufacturing constraints are significant. Imported devices are expensive, and the dependence on them undermines sustainability. For example, a review on developing pediatric cardiac programs emphasised that due to the high costs of importing equipment, treatment is limited [22].

Brazil has reported isolated cases of using MCS in pediatric patients, including the first pediatric HeartMate 3 implantation in 2021, but such examples remain exceptions [23]. ECMO, though occasionally available, is sporadically utilized and often limited to large urban centers. For the vast majority of children in LMICs, there is no safety net between progressive heart failure and death.

## 5. Success Stories and Emerging Programs 

Despite these challenges, there are promising developments across some LMICs. India has emerged as one of the few LMICs with a significant pediatric transplant program, with survival outcomes comparable to LMIC benchmarking standards. In a recent series, 90-day survival was over 85%, with follow-up extending to 10 years [12]. Iran has also shown hopeful outcomes, as evidenced by a study done in a center in Iran where 225 pediatric heart transplants were performed from 2012 to 2021. In this study, the 1-year, 3-year, and 5-year survival rates were 85.7%, 79.7%, and 73.9% respectively [15]. Brazil has also taken incremental steps, with isolated pediatric transplants supported by MCS devices despite economic and infrastructural barriers [23]. By utilising LVADs, the bridge to heart transplantation is now possible. These successes highlight what is possible when local expertise, institutional commitment, and government support are aligned.

However, despite these successes, the majority of LMIC do not have a sustainable pediatric heart transplant program. In Africa, only a limited number of countries have reported isolated cases of pediatric heart transplants [24]. This emphasizes the urgent need for investment and collaboration.

## 6. The Way Forward 

**Fig. 1.**
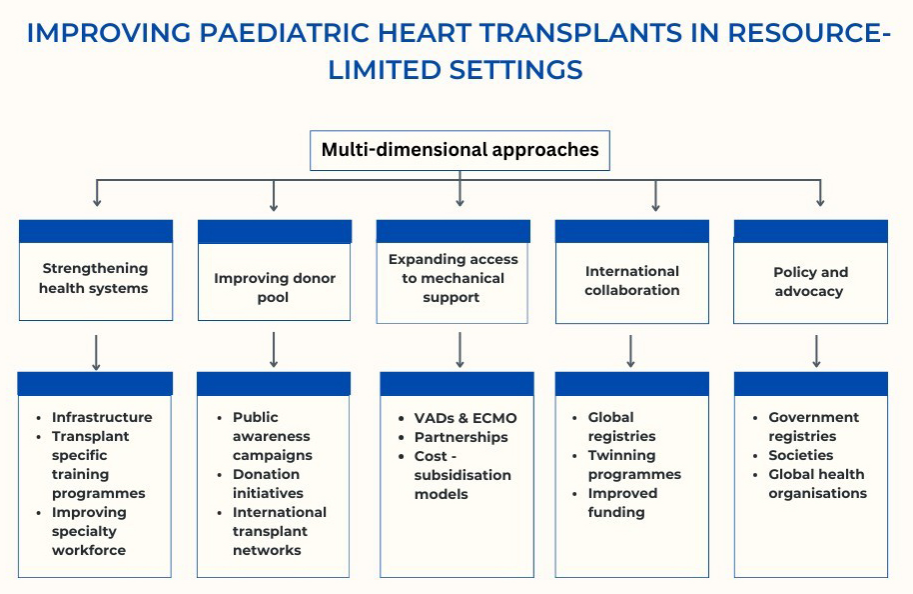
**Summarises the proposed strategies and maps the steps that need to be taken to improve pediatric heart transplants in LMIC**. ECMO, extracorporeal membrane oxygenation; VADs, ventricular assist devices.

Addressing this silent crisis requires coordinated action at multiple levels:

1. Strengthening health systems: Investment in pediatric cardiac surgery infrastructure, training programs, and multidisciplinary teams is essential. Without robust surgical and intensive care capacity, transplantation cannot be sustained. In Nigeria, the cost for open heart surgery for pediatric congenital heart disease ranged from $ 6000– $ 11,000, which is similar to other LMIC [25]. These costs are restrictive for families of patients in LMIC, as these staggering costs exceed their average household incomes. This signifies the urgent need for sustainable funding mechanisms to ensure children have access to life-saving treatment regardless of where they live.
2. Promoting organ donation: pediatric-specific donation initiatives, public awareness campaigns, and improved organ transport networks are urgently needed. Innovative solutions, such as regional organ-sharing collaborations, could mitigate geographic barriers.
3. Expanding access to mechanical support: Partnerships with industry and non-governmental organizations could make VADs and ECMO more accessible. Local manufacturing or cost-subsidization models may help overcome financial barriers.
4. International collaboration: Global registries, twinning programs — partnerships between two institutions that share knowledge and resources to achieve a common goal — and targeted funding mechanisms should be established to support centers in LMICs.
5. Policy and advocacy: Governments, professional societies, and global health organizations must prioritize pediatric transplantation within broader cardiovascular health agendas. Financial sustainability must be a key component of national health strategies such as insurance schemes and non-governmental organization (NGO) partnerships. Alongside these ethical and regulatory frameworks, there must also be the promotion of fairness in cross-border organ sharing. These frameworks must guarantee that wealthier patients do not disproportionately receive access to care and must ensure that there is no exploitation of the poor. Without political will, the current inequities will persist (Fig. 1 ).

## 7. Conclusion 

Pediatric heart transplantation remains a silent crisis in resource-limited settings. While thousands of children in HICs receive lifesaving transplants each year, countless others in LMICs die without access to even basic palliative options. Success stories from India and Brazil prove that progress is possible in LMICs, but without concerted global action, these will remain isolated exceptions. The time has come to recognize pediatric cardiac transplantation as not only a global health challenge but a moral imperative. No child, regardless of geography, should be left to die waiting for a chance at life.
